# Metabolome restructuring reveals distinct virocell infection strategies in bacteriophage–host interaction

**DOI:** 10.1093/ismeco/ycag223

**Published:** 2026-08-04

**Authors:** Sumudu Rajakaruna, Cristina Howard-Varona, Marion Urvoy, Roya AminiTabrizi, Christian Ayala-Ortiz, Marissa Gittrich, Natalie Solonenko, Marie Burris, Courtney Sanderson, Cara Noel, Jonathan Leopold, Lillyanna Quillin, Kush Doshi, Lawrence R Walker, Matthew B Sullivan, Malak M Tfaily

**Affiliations:** Department of Environmental Science, University of Arizona, Tucson, AZ 85721, United States; BIO5 Institute, University of Arizona, Tucson, AZ 85719, United States; Department of Microbiology, The Ohio State University, Columbus, OH 43210, United States; Center of Microbiome Science, The Ohio State University, Columbus, OH 43210, United States; Department of Microbiology, The Ohio State University, Columbus, OH 43210, United States; Center of Microbiome Science, The Ohio State University, Columbus, OH 43210, United States; Department of Environmental Science, University of Arizona, Tucson, AZ 85721, United States; Department of Environmental Science, University of Arizona, Tucson, AZ 85721, United States; Department of Microbiology, The Ohio State University, Columbus, OH 43210, United States; Center of Microbiome Science, The Ohio State University, Columbus, OH 43210, United States; Department of Microbiology, The Ohio State University, Columbus, OH 43210, United States; Center of Microbiome Science, The Ohio State University, Columbus, OH 43210, United States; Department of Microbiology, The Ohio State University, Columbus, OH 43210, United States; Department of Microbiology, The Ohio State University, Columbus, OH 43210, United States; Center of Microbiome Science, The Ohio State University, Columbus, OH 43210, United States; Department of Microbiology, The Ohio State University, Columbus, OH 43210, United States; Department of Microbiology, The Ohio State University, Columbus, OH 43210, United States; Department of Microbiology, The Ohio State University, Columbus, OH 43210, United States; Department of Biochemistry, The Ohio State University, Columbus, OH 43210, United States; Analytical and Biological Mass Spectrometry Facility, University of Arizona, Tucson, AZ 85721, United States; Department of Microbiology, The Ohio State University, Columbus, OH 43210, United States; Center of Microbiome Science, The Ohio State University, Columbus, OH 43210, United States; Department of Civil, Environmental and Geodetic Engineering, The Ohio State University, Columbus, OH 43210, United States; Department of Environmental Science, University of Arizona, Tucson, AZ 85721, United States; BIO5 Institute, University of Arizona, Tucson, AZ 85719, United States

**Keywords:** virocell, bacteriophage-host interactions, untargeted metabolomics, metabolite annotation, *Cellulophaga baltica*, infection efficiency, temporal metabolic dynamics, marine heterotrophic bacteria, metabolic reprogramming, multivariate metabolomics

## Abstract

Viral infection transforms microbial cells into metabolically reprogrammed “virocells,” yet the metabolic architecture underlying this transition remains poorly resolved. Here we investigate how bacteriophages reshape host metabolism in *Cellulophaga baltica* across time and phage types, uncovering coordinated metabolic reorganization during infection that includes pathway-level patterns consistent with membrane remodeling, amino-acid recycling, and redistribution of cellular resources. Temporal analyses further distinguished infection strategies: efficient phages produced structured, phased metabolic shifts indicative of controlled host reprogramming, whereas inefficient infection triggered abrupt metabolic collapse. These results suggest that the organization and timing of host metabolome restructuring represent defining features of the virocell state and may reflect underlying viral life-history strategies. These biological patterns were made accessible by an integrative annotation framework that combines previously validated computational tools, substantially expanding interpretable metabolite coverage beyond conventional approaches. This approach reveals coherent infection signatures that would otherwise remain hidden, demonstrating how expanded metabolite interpretability can uncover functional principles of virus–host interactions. The analytical strategy presented here is readily transferable to other virus–host systems and complex microbial communities, providing a path toward mechanistic interpretation of untargeted metabolomic data in microbial ecology.

## Introduction

Microorganisms are central drivers of ecosystem processes, mediating nutrient cycling, carbon turnover, and environmental resilience [1–3]. Among the forces shaping microbial metabolism, viruses exert particularly strong influence. By infecting and reprogramming host cells, bacteriophages alter cellular physiology, redirect metabolic fluxes, and reshape the biochemical outputs of microbial populations [4–8]. Virus-infected cells, now widely referred to as *virocells*, differ fundamentally from uninfected cells in both their functional state and metabolic identity [9–11]. Therefore, understanding how viral infection restructures host metabolism is critical for linking molecular-scale interactions to broader ecological processes.

Phage-driven metabolic reprogramming arises through multiple mechanisms. Viral auxiliary metabolic genes, altered host gene expression, and enzyme modulation can redirect carbon and energy flow toward virion production while suppressing competing cellular processes [4–8]. Such changes may alleviate metabolic bottlenecks, enhance precursor availability, and alter redox balance or membrane composition. While these strategies have been documented in several phage systems, most studies focus on individual phage–host pairs, limiting our ability to distinguish general infection responses from phage-specific strategies (Supplementary Table 1). Moreover, comparatively little is known about how different phage types or infection efficiencies shape host metabolic trajectories, particularly in marine heterotrophic bacteria where viruses are abundant and ecologically influential [12–17].

Metabolomics offers a direct route to probing these infection-induced metabolic shifts, as it captures the small molecules that reflect ongoing biochemical activity. In particular, untargeted LC–MS/MS approaches can detect thousands of molecular features per sample [18–22], offering system-wide views of cellular metabolism. However, interpretation remains constrained by a persistent annotation bottleneck: typically only 5%–20% of detected features can be confidently identified [23, 24]. This limitation reduces the biological insight that can be extracted from otherwise information-rich datasets and is particularly restrictive in microbial systems, where metabolite diversity often exceeds available reference libraries [25–27].

Recent advances in computational annotation, machine learning, and multivariate statistics offer opportunities to partially overcome this constraint [28–37]. Critically, several such tools have been independently developed and validated, including CMMRT for probabilistic retention-time-guided annotation and SIRIUS with CANOPUS for in silico structural and chemical class inference; this study applies them in combination for the first time in a virocell metabolomics system. Rather than relying solely on library matches, integrating these established analytical approaches can expand the interpretable metabolite space and improve detection of coordinated metabolic shifts. When coupled with ecological and statistical frameworks capable of capturing multivariate and temporal structure, such approaches can help reveal infection-driven metabolic programs even when individual metabolite identities remain putative. Hence, here, we apply these tools in combination to a controlled virocell model system involving the marine bacterium *C. baltica* strain 18 infected independently by three previously characterized bacteriophages: the efficient dsDNA phage phi18:1, the efficient ssDNA phage phi18:4, and the inefficient dsDNA phage phi38:1 [12–17]. This system enables direct comparison of host metabolic responses across phages that differ in genome type, infection efficiency, and replication dynamics. We hypothesized that these differences would produce both shared infection signatures and phage-specific metabolic programs, potentially reflected in distinct temporal trajectories of metabolic reprogramming.

Using untargeted metabolomics combined with integrative annotation, multivariate analysis, and time-resolved modeling, we characterize how phage infection reshapes host endometabolomes across these systems. Our results reveal four major biological patterns: 1) a conserved metabolic response shared across infections, 2) phage-specific metabolic signatures, 3) metabolic trajectories associated with infection efficiency, and 4) distinct temporal coordination of host metabolic reprogramming.

Together, these findings reveal both conserved and divergent metabolic strategies during viral infection, demonstrating that metabolome architecture and temporal coordination are defining features of the virocell state. By combining established annotation and statistical frameworks to expand interpretable metabolite coverage, this study enables biological investigation of virus–host interactions at a depth not previously achievable with conventional untargeted metabolomics workflows.

## Materials and methods

### *C. baltica* growth experiments and sampling

Time-resolved experiments were conducted as in prior work [9, 12, 14]. Phages were added to *C. baltica* #18 at an MOI of 2, 3, and 5 for phi18:1, phi18:4, and phi38:1, respectively, and allowed to adsorb for 10 min (phi18:4) or 15 min (phi18:1 and phi38:1) depending on their previously measured adsorption rate [15, 16]. Cells were grown to mid-exponential phase, then diluted to 1x10^8^ CFU/ml in a total volume of 80 ml of marine Luria Bertani (MLB). All samples were then diluted 10-fold to prevent further phage adsorption and synchronize the infections to improve our ability to detect biological changes over the stages of phage infection. An uninfected control condition was prepared similarly and amended with marine sodium magnesium phage buffer (450 mM NaCl, 50 mM MgSO_4_, 50 mM Tris, pH 7.5). Samples were taken at various points post-dilution and pre-lysis for phi18:1 and phi18:4 (0-, 15-, 30-, and 45-min post-dilution), or longer for phi38:1 (0, 15-, 30-, 120-, and 300-min post-dilution), with the uninfected control sampled at each corresponding time point. Timepoints were selected based on previously characterized infection kinetics for each phage [15, 16] and confirmed to be pre-lysis for phi18:1 and phi18:4 through concurrent CFU and PFU plating at each timepoint, with phi18:4 showing minimal phage production (~1% of peak free phage counts) at T45. For phi38:1, all metabolomics timepoints fall within its prolonged latent period, ensuring that observed metabolic dynamics reflect active infection states rather than post-lytic artifacts. Although metabolomic samples were also collected at 300 min for phi38:1, these samples failed quality-control assessment due to technical issues during metabolomic processing and were excluded from downstream analyses. Each time point for all conditions had four biological replicates. Total and free plaque forming units (PFUs) and colony forming units (CFUs) were plated for each sampling experiment to monitor phage production and cell lysis. For endometabolome, two 35 ml samples per biological replicate were centrifuged (10 000 g for 10 min at 4°C) and supernatants were discarded. Pellets were resuspended in 0.5 ml of phosphate buffered saline (PBS), combined, washed again in 1 ml PBS, flash frozen in liquid nitrogen, and stored at −80°C.

### Endometabolomic extractions and LC–MS/MS

Cell pellets were resuspended in 100 μl ddH_2_O, transferred to glass vials and subjected to 3 freeze–thaw cycles to facilitate cellular disruption. Probe sonicator (Bio-Gen PRO200) was used to agitate samples on 5–10 second pulses up to 5 mins while the vials remained on ice, to ensure complete cellular breakdown and the release of endometabolites. MPLEx extraction of the samples was carried out using a 3:8:4 ratio of ddH_2_O:CHCl_3_:MeOH, accounting for the 100 μl ddH_2_O added before [38]. The samples were briefly vortexed and incubated overnight at 4°C to facilitate phase separation. Metabolite containing MeOH layer was carefully removed and transferred to two vials each containing equal volumes for reverse-phase (RP) and hydrophilic interaction liquid chromatography (HILIC) modes. Vials were dried down using vacuum-centrifuge (Eppendorf Vacufuge Plus) and stored at −80°C till processing. On the day of processing, RP vials were reconstituted using 400 μl of 80:20 mixture of ddH_2_O:Methanol solution and HILIC vials were reconstituted using 400 μl of 50:50 Acetonitrile: ddH_2_O. Pooled controls were generated for each mode separately by mixing 10 μl from each sample. Pooled QC-based area corrections were implemented to normalize ionization levels across the entire experiment and address various systematic biases commonly encountered in large-scale metabolomics analyses. Metabolite extracts were analyzed using liquid chromatography electrospray ionization tandem mass spectrometry (LC-ESI-MS/MS) on a Thermo Vanquish UHPLC system, coupled with a Thermo Exploris 480 Orbitrap mass spectrometer. Both RP and HILIC phases were utilized during the analysis. A 1 μl sample was introduced into the column, with the following gradient elution conditions: for RP, a gradient transitioned from 99% mobile phase A (0.1% formic acid in H_2_O) to 95% mobile phase B (0.1% formic acid in methanol) over 16 minutes; and for HILIC, the gradient was from 99% mobile phase A (0.1% formic acid, 10 mM ammonium acetate, 90% acetonitrile, 10% H_2_O) to 95% mobile phase B (0.1% formic acid, 10 mM ammonium acetate, 50% acetonitrile, 50% H_2_O). Both columns were maintained at 45°C, with a flow rate set to 300 μl/min. For RP LC, a Waters ACQUITY Premier HSS T3 column (1.8 μm, 2.1 mm × 150 mm) was employed, while for HILIC LC, an ACQUITY UPLC BEH HILIC amide column (1.7 μm, 2.1 mm × 150 mm) was used. The gradient was consistent across both RP and HILIC chromatography conditions.

### Metabolite data collection

Full MS scan data were acquired with a resolving power of 120 000 full width at half maximum (FWHM) at m/z 200, covering a scan range from m/z 65 to 975. The automatic gain control (AGC) target was set to 50%, and the maximum injection time was capped at 100 ms. For data-dependent acquisition (DDA, dd-MS2), product ion spectra were collected at a resolving power of 30 000 FWHM at m/z 200, with an AGC target of 50% and the maximum injection time set to auto. The isolation width was fixed at 1.2 m/z, and higher-energy collisional dissociation (HCD) was applied with collision energies of 20%, 40%, and 80%. The top 10 most intense peaks from each MS1 scan were selected for the DDA analysis. Samples were analyzed in both positive and negative ionization modes. Raw spectra processing including peak picking, alignment, correspondence, and metabolite annotations were performed using Thermo Compound Discoverer 3.3, applying the previously specified parameters [39].

### Metabolite annotation

CMMRT and SIRIUS with CANOPUS are independently published and validated tools [35, 36]; here we apply them in combination for the first time in a virocell metabolomics context alongside library-based approaches to maximize biologically interpretable coverage. We used a multi-tool annotation integration framework designed to expand the interpretable metabolite space**,** summarized in Supporting Figs 1–3. Spectra for both RP and HILIC modes were first annotated for Level 1 identified compounds (matching accurate mass, MS2 spectral similarity, and retention time against authentic standards) and Level 2 annotations (matching accurate mass and MS2 spectral similarity to library spectra), as per the standards described by Metabolomics Standards Institute [40], using Compound Discover (version 3.3). Additionally, RP and HILIC processed spectra were screened for documented in-source fragments to reduce the statistical burden of duplicate entries [41, 42]. The verification of in-source fragments was achieved using the criteria of high correlation >0.85 with mass difference between features corresponding to the mass of known adducts.

### Database curation and biological relevance filtering

This filtered RP dataset was subjected to CMMRT (Chromatographic Method Meta-learned Retention Time) algorithm, which combines retention time prediction using a deep learning model and retention time projection across chromatographic methods using a Bayesian meta-learning model that requires only a few known metabolites [35]. Accordingly, we used 10 Level 1 identified compounds as standards to obtain meta-learned retention time corrections for the remaining compounds, which were subsequently mapped along with corresponding MS1 to METLIN Small Molecule Retention Time (SMRT) database [43] using mass tolerance of ±5 ppm and retention time tolerance of ±30 seconds to obtain a list of probabilistic annotations as per the guidelines provided [35]. All SMRT-derived annotations underwent systematic manual curation to ensure biological relevance to marine bacterial systems. The RT projection model consisted of a deep neural network (DNN) that was trained using the METLIN-SMRT dataset with cosine annealing warm restarts and stochastic weight averaging. This DNN model had a mean error of 39.2 ± 1.2 s, and median absolute error of 17.2 ± 0.9 s. The retention time projection model consisted of a Bayesian meta-learning that was trained using four different CMs from the PredRet database [44], and a radial basis function kernel. Both models were adapted into a Dash app [45], that uses as an input a list of unknown metabolites with their m/z and retention time, as well as a list of at least 20 known metabolites with their PubChem ID. Once finished the app returns potential candidate annotations ranked based on z-scores.

### Annotation performance assessment

We evaluated our predicted candidate annotations against existing Level 1 metabolites in our dataset, which achieved 69% correct identification accuracy when validated against known Level 1 metabolites in our dataset, in agreement with the published classification accuracy of 68% reported for CMMRT algorithm [35]. This validation was performed using only the subset of metabolites with confirmed Level 1 identifications and does not guarantee similar accuracy for all predicted annotations. However, computational annotations represent putative identifications requiring experimental validation for definitive confirmation. Single candidate metabolite predictions from CMMRT were considered as Level 3 annotations (putative identification based on MS1 and predicted retention time), while multi-predictions (up to 3) were manually curated and assigned to Level 3 using the following systematic priority criteria (Supporting Fig. 3C); 1) exclusion of synthetic pharmaceuticals and non-biological compounds, 2) verification that a candidate was not already present among annotated Level 1–2 metabolites to avoid redundancy, 3) prioritization of metabolites present in Kyoto Encyclopedia of Genes and Genomes (KEGG) pathways for *C. baltica* #18 [46], 4) selection of compounds previously reported in biological systems of relevance (microbial systems based on literature review of HMDB, MetaCyc, and peer-reviewed publications). If these criteria could not resolve a definitive candidate amongst the multi-prediction, additional steps were employed as follows, where each candidate’s chemical classes were assessed using ClassyFire algorithm [47]. If all candidates belong to a particular chemical class hierarchy, the compound was denoted as “undefined” followed by the bottommost common hierarchical order (e.g.: *undefined dipeptide x*) (step 05). If candidates could not be resolved using chemical class hierarchy, they were denoted as an “undefined” followed by the highest chemical hierarchies obtained (e.g.: undefined Lipids and lipid-like molecule or organoheterocyclic compound x) (step 06). This systematic curation process significantly reduces false positive annotations while maintaining comprehensive coverage of biologically plausible metabolites for marine bacterial systems.

Molecular classes of the remaining unannotated RP metabolites and HILIC metabolites were predicted with CANOPUS, based on fragmentation trees built using SIRIUS where top candidate class based on Tanimoto score was used to assign chemical class of each metabolites [36]. These were also considered Level 3 metabolites (MS1 and in silico predicted class level annotations). Detailed breakdown of all the candidates from CMMRT and multi-candidate selection is provided elsewhere (Workbook S1).

### Data analysis

RP and HILIC datasets were normalized using log10 transformation and median scaled as done before [39]. All downstream analyses were performed in R (version 4.3.0) [48] and Orange (version 3.37.0) [49] platforms, independently for RP and HILIC datasets. Ordination analyses were performed using R(vegan) package [50], through Principal Coordinate Analysis (PCoA) using Bray-Curtis distances. Significance of constrained variables in PCoA ordinations were assessed using permanova R(adonis2) function. Discriminatory metabolites between uninfected control and each virocell were obtained independently using orthogonal projections to latent structures discriminatory analysis (OPLS-DA) using R(*ROPLS*) package [51], with VIP > 1 cutoff and subsequent cross-validation using independent BH corrected t-tests (< 0.05) as done before [52]. OPLS model performances are provided elsewhere (Supplementary Table 2). Results were evaluated using a diVENN plot [53], to highlight unique and shared discriminatory metabolites which are significantly different for each virocell compared to their uninfected counterparts. Independently, logistic regression algorithm with lasso regularization was used to obtain predictive metabolites of each virocell infection, irrespective of the host, using Orange [49] as done before [54]. These predictive features were cross compared with OPLS discriminatory results to filter predictive endometabolites of each virocell infection that are simultaneously also significantly different compared to the uninfected host; thereby being strong candidate biomarker metabolites representing putative signatures of each virocell infection. KEGG pathway matching was performed in R(*KEGGREST*) and manually curated to accommodate alternative names of each metabolite checked [46]. Endometabolomic temporal trends were evaluated using a combined approach of Principal Response Curves analysis (PRC) [55], linear model (LM) [56], and Dynamic Time Warping (DTW) in R (*VEGAN, DTWCLUST*) [57]. Briefly, global temporal trends of each virocell were evaluated using PRC as done before [58], to obtain top 2% of temporally changing metabolites. Only the RP dataset was used to obtain global temporal trends, with the top 2% of metabolites from each PRC analysis (n = 39 per virocell type) selected for subsequent analysis. This number was deemed sufficient to elicit clear temporal clusters while minimizing the potential for spurious signatures. RP metabolites were independently evaluated by a LM using *P* < .05, BH adjusted and log2FC >1 criteria, having compared timepoint counterparts to the uninfected control (Workbook S2). These top globally changing temporal metabolites of each virocell progression (PRC) that are significantly different at least at a single timepoint to its uninfected counterpart (LM) were used for temporal clustering to obtain top endometabolomic trends observed for each virocell (Workbook S2). These were used in the DTW algorithm to obtain temporal clusters of each virocell progression, using average distances <1.2 as a number of clusters threshold. All plots were generated in R unless specified otherwise and combined into compound figures in Adobe illustrator®.

### Integrative annotation framework

To maximize biologically interpretable signals from untargeted LC–MS/MS data, we developed an integrative annotation framework combining previously validated computational tools alongside dual-column chromatography and in-source fragment removal (Supporting Figs 1–3). Combining RP and HILIC broadened metabolite class coverage beyond single-column approaches [39] (Supporting Fig. 3A,B). Removal of known in-source fragments reduced technical noise and improved reliability of downstream comparisons [41, 42]. For annotation, we combined CMMRT for probabilistic retention-time-guided annotation of RP data and SIRIUS/CANOPUS for chemical class prediction across both chromatographic modes [35, 36], applying them together in a virocell metabolomics context for the first time. Together, these approaches generated MSI Level 3 annotations, with all predicted annotations subsequently curated using organism-specific biological relevance criteria to reduce implausible assignments (Supporting Fig. 3). Applied to our dataset, this framework detected 2814 features (1910 RP; 904 HILIC), with minimal overlap between chromatographic modes (n = 9), confirming that each mode captured distinct metabolite classes. Conventional annotation approaches alone identified 36.0% of RP and 4.7% of HILIC features (25.9% overall), whereas the integrative framework increased interpretability to 89.9% for RP and 25.6% for HILIC (69.3% total) (Supporting Fig. 3). It is important to note that increased interpretability does not imply definitive compound confirmation, Level 3 annotations remain putative and require targeted validation. The multivariate and temporal patterns reported here are derived from the statistical structure of the full feature space rather than the identity of any individual metabolite; the annotation framework therefore provides a biological lens for interpreting these patterns rather than a claim of confirmed identities.

## Results

### Phage identity and infection stage drive distinct virocell metabolic trajectories

**Figure 1:**
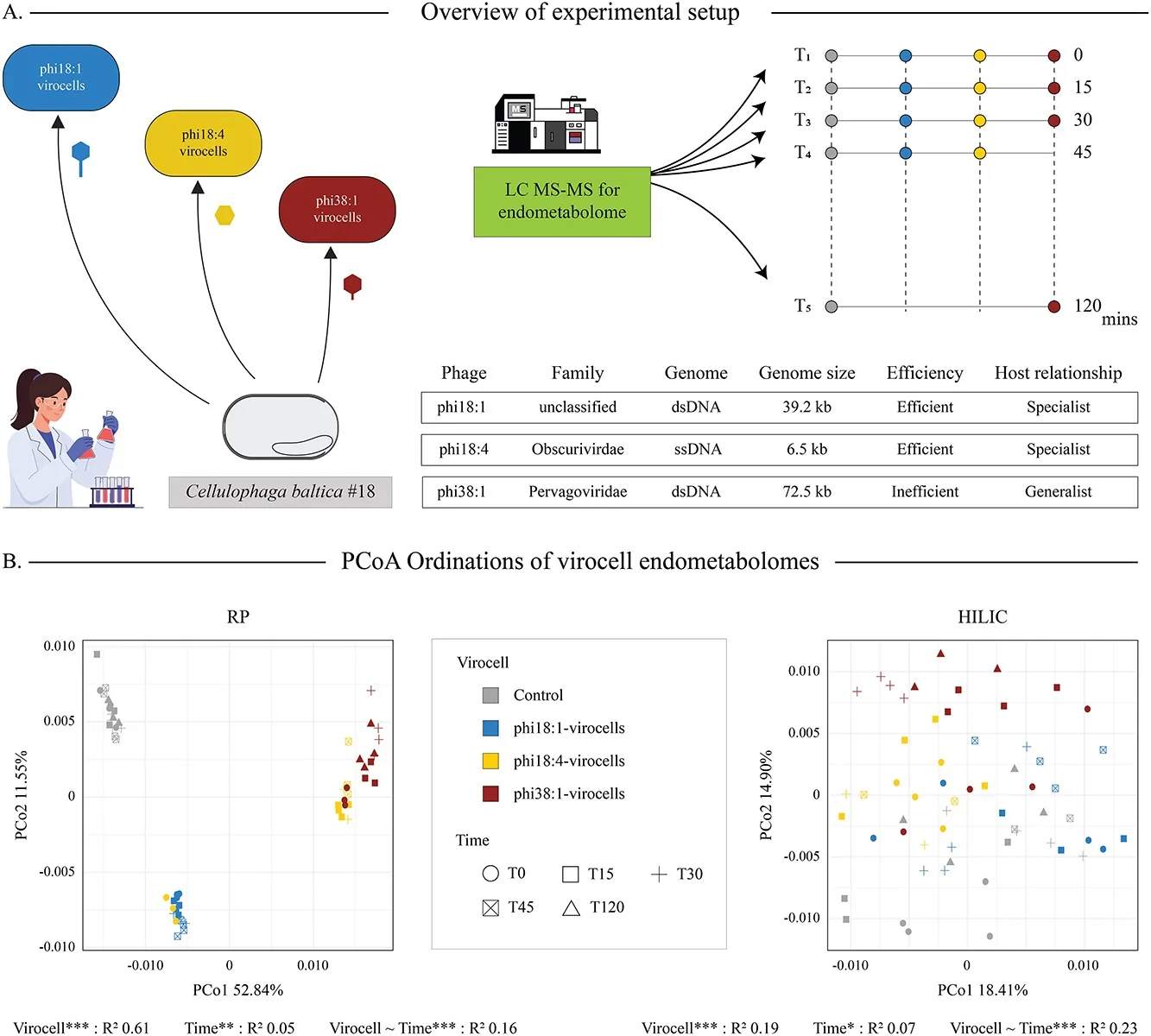
Overview of the experimental setup and endometabolome ordinations. Panel A depicts the sampling overview. *Cellulophaga baltica* strain #18 was independently infected with three distinct phages, dsDNA phage phi18:1, ssDNA phage phi18:4, and dsDNA phage phi38:1. Temporal sampling was performed across infection progression to capture virocell endometabolomes. Uninfected controls were included for comparison. Samples were analyzed using LC–MS/MS with both RP and HILIC chromatographic modes. Panel B shows PCoA plots based on Bray–Curtis dissimilarity that illustrate global shifts in metabolomic profiles, analyzed separately for RP and HILIC datasets. Ordinations were conducted as part of the enhanced framework, integrating multi-tool annotation with multivariate statistical analysis. Clear clustering by virocell identity and time reflects phage-specific and temporally dynamic metabolic reprogramming. Contributions of phage identity and time to variation were evaluated using PERMANOVA (*P* < .05); significance values are indicated below each plot.

Using the integrative annotation framework described in Methods, we examined how phage infection reshapes the host endometabolome across three distinct virocell systems, revealing coordinated metabolic reorganization at both global and pathway levels. To determine whether expanded metabolite coverage reveals biologically meaningful infection dynamics, we examined global endometabolome structure using ordination approaches commonly applied in microbial ecology. PCoA based on Bray–Curtis distances revealed that both infecting phage and time strongly structured metabolite profiles in RP and HILIC datasets (PERMANOVA, *P* < .05; Fig. 1B). Because this approach captures community-level structure rather than relying on individual metabolite identities, it is particularly suited for detecting system-level metabolic reprogramming during infection [59].

Across datasets, infected samples separated clearly from uninfected controls, indicating that phage infection consistently reshapes host metabolic states. However, trajectories differed among phages. In RP data, virocells infected by the efficient dsDNA phage phi18:1 and the ssDNA phage phi18:4 initially followed similar metabolic profiles, consistent with comparable early infection dynamics. Over time, however, phi18:4-infected cells shifted toward the metabolic state observed in the inefficient dsDNA phage phi38:1 infection. This divergence suggests that infection outcome is not determined solely by early host takeover, but also by the timing and coordination of metabolic restructuring, with ssDNA infection exhibiting a delayed or less synchronized metabolic transition. It may also reflect a constraint of small phages that do not encode their own replication machinery and heavily rely on host metabolism for replication. Indeed, despite achieving efficient infection, phi18:4’s small genome (6.5 kb) may be insufficient to sustain the coordinated host reprogramming observed in phi18:1 beyond the initial takeover phase, potentially resulting in metabolic drift toward a trajectory more closely resembling the declining signature of phi38:1. While speculative, this interpretation is broadly consistent with the known differences in burst size between these phages (90 for phi18:1 vs. 41 for phi18:4; ref 15), suggesting that sustained metabolic coordination may be a prerequisite for efficient progeny production. Furthermore, the PERMANOVA R^2^ values provide additional biological insight: virocell identity explained a substantially larger proportion of variance in RP data (R^2^ = 0.61) compared to HILIC data (R^2^ = 0.19), while the virocell × time interaction term contributed more equally across modes (R^2^ = 0.16 and 0.23, respectively). This pattern suggests that phage-specific metabolic signatures are carried preferentially by nonpolar metabolite classes captured in RP mode, whereas temporal infection dynamics are comparably reflected across both polar and nonpolar chemical spaces.

### Shared and phage-specific metabolic signatures distinguish virocell states

Having established that infection drives distinct metabolic trajectories, we next asked which metabolites contribute to these separations. To identify features underlying group differentiation, we applied OPLS-DA, a multivariate framework capable of detecting coordinated metabolic shifts across large feature sets [51]. Unlike univariate approaches that evaluate metabolites independently, this method captures covariance structures, allowing detection of coordinated biochemical responses associated with infection.

**Figure 2:**
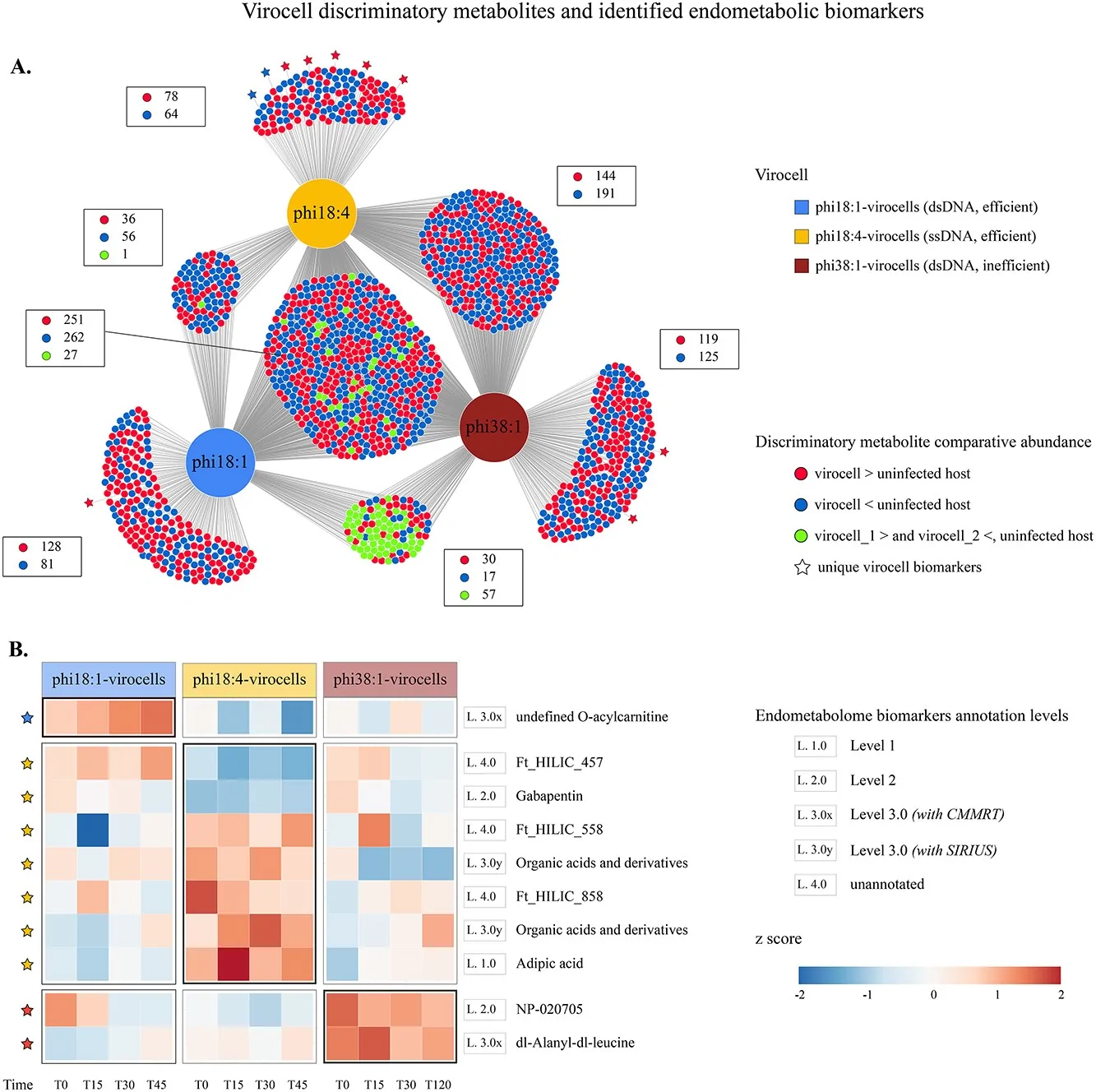
Identification of discriminatory and candidate biomarker metabolites across C. baltica virocells using multivariate statistical analysis. Panel A presents a DiVenn visualization of discriminatory metabolites identified through independent OPLS-DA analyses comparing each virocell (phi18:1, phi18:4, phi38:1) to the uninfected control. Each node represents a metabolite that passed dual statistical thresholds (VIP ≥ 1.0 and adjusted *P* < .05), with edges connecting nodes detected in multiple OPLS-DA comparisons. The number of edges thus denotes the level of overlap. Nodes with single edges represent metabolites unique to a single virocell (isolated islands), two way edges indicate shared metabolites between two virocells, and three way overlaps denote metabolites shared across all virocells. Metabolite comparative abundance relative to controls is indicated as shown in the figure legend, distinguishing metabolites enriched in virocells, metabolites depleted in virocells, and metabolites with inconsistent trends, meaning shared between multiple comparisons where one shows enrichment and the other depletion. Boxed inserts indicate the number of metabolites in each unique or shared set, with breakdowns for each intersection. While this panel depicts all discriminatory metabolites, those also predicted as candidate biomarkers, that is, predictive of virocell type via logistic regression, are additionally marked with stars. These include putative identifications requiring experimental validation. Panel B shows a heatmap of temporal abundance profiles for these candidate biomarker metabolites. Candidate biomarkers (marked with stars and requiring experimental validation for definitive confirmation) were defined as metabolites that are [1] significantly different in virocells versus controls that are also unique (isolated islands) and are [2] independently discriminatory between virocell types via logistic regression analysis, without the host influence. These represent computational predictions based on Level 3 annotations.

To prioritize robust biological signals, we used a conservative dual-threshold strategy in which discriminatory metabolites were required to have both a VIP score ≥ 1.0 and an adjusted *P* < .05 from independent univariate testing. This approach identified extensive infection-associated metabolic restructuring, with 946 discriminatory metabolites detected in phi18:1 virocells, 1110 in phi18:4, and 1223 in phi38:1 infections (Fig. 2). These features represent the biochemical drivers associated with the infection-specific trajectories observed in the ordination analysis.

Cross-comparison revealed that 540 discriminatory metabolites, approximately 20% of significant features, were shared across all three infections, and strikingly, 95% of these showed consistent abundance trends. This conserved response suggests that virocells may exhibit a core metabolic program triggered by phage infection, independent of genome type or infection efficiency. Such metabolites represent strong candidates for a core virocell metabolic program, a conserved biochemical response to viral takeover that may operate independently of phage genome type or infection efficiency [9–11]. Importantly, the directional consistency of 95% of these shared metabolites suggests this conserved response is not statistical noise but reflects structured, coordinated host reprogramming common to virocell states.

Although conserved infection responses have been observed in virocell transcriptomic [12, 14] and lipidomic datasets [60, 61], they remain uncommon in metabolomics (Supplementary Table 1). For example, a prior study of *Pseudomonas aeruginosa* infected by 5 lytic phages reported only 2.4% shared metabolites across infections [62]. The broader shared metabolic signal detected here suggests that deeper metabolite coverage can reveal conserved infection biology that may previously have been obscured. It is worth noting that the prior study reporting only 2.4% shared metabolites across *P. aeruginosa* infections involved five phages and relied on library-validated metabolites alone [62]. While host identity, phage diversity, and annotation depth all likely contribute to differences between studies, the broad conserved metabolic signal observed here across three genomically distinct phages that lack detectable shared genes based on pairwise BLASTN comparisons of predicted coding sequences (EasyFig analysis), strengthens the case that a core infection-responsive metabolic program may exist in *C. baltica* virocells, and possibly more broadly. Whether such conserved signatures extend across host taxa remains an important open question that our findings motivate. Together, these findings indicate that virocell metabolism is shaped by both universal infection responses and phage-specific metabolic programs, underscoring the value of system-level metabolomics for resolving infection ecology.

### Integrated statistical learning highlights candidate metabolic markers of infection states

To determine whether infection-associated metabolic shifts could be distilled into specific biochemical indicators, we next searched for metabolites that were not only discriminatory but also consistently predictive of infection identity. Rather than treating all discriminatory metabolites equally, we prioritized features that may represent stable biochemical markers of virocell states across conditions.

To achieve this, we combined multivariate discrimination with predictive modeling in a two-stage framework (Fig. 2B). Orthogonal Projections to Latent Structures–Discriminant Analysis identifies metabolites contributing most strongly to infection-driven separation [51], whereas lasso-regularized logistic regression independently selects metabolites that best predict virocell identity regardless of shared host background [63]. By intersecting outputs from these complementary approaches, we prioritized metabolites that were both statistically robust and uniquely predictive of infection state, thereby enriching for biologically interpretable candidates while minimizing false positives. These features should be viewed as hypothesis-generating candidates for future validation rather than definitive biomarkers.

This integrative analysis yielded 10 candidate metabolic markers across infections (Fig. 2): seven associated with phi18:4 virocells, one with phi18:1, and two with phi38:1. Three candidates corresponded to high-confidence annotations (Levels 1–2), four were supported by computational annotations within the expanded framework, and three remain chemically unresolved.

The sole phi18:1-associated candidate was putatively classified as an enriched O-acylcarnitine species (Level 3, CMMRT annotation). Acylcarnitines have been implicated in fatty acid transport for beta-oxidation, serving as a key route to ATP generation under conditions of sustained energetic demand [64–67]. Their enrichment during phi18:1 infection is therefore biologically plausible in the context of phi18:1’s infection kinetics: with a latent period of ~65 minutes and the largest burst size of the three phages, i.e. 90 [15], phi18:1 imposes a prolonged replication demand on the host. O-acylcarnitine enrichment is therefore biologically consistent with sustained host energy mobilization required to support this extended, productive replication cycle, a metabolic strategy that would be less necessary in phages with shorter latent periods or lower burst sizes.

Phi18:4 virocells had more metabolite markers, and among higher-confidence annotations, gabapentin (Level 2) was depleted and adipic acid (Level 1) was enriched. Adipic acid is a six-carbon dicarboxylic acid involved in fatty acid and lysine catabolism, and its enrichment may potentially reflect altered carbon flux during the rapid metabolic takeover characteristic of this ssDNA phage [15]. Gabapentin depletion is less straightforward to interpret in a marine bacterial context, though structural analogs of gamma-aminobutyric acid have been reported in bacterial stress responses. The remaining phi18:4 candidates derive from chemical class-level predictions and are intentionally not interpreted at the individual compound level given current annotation uncertainty. Notably, phi18:4 is the only phage in this study whose infection success has been shown to be specifically dependent on host lipid composition in this same *C. baltica* system; phage resistance arising from serine synthesis pathway mutations was shown to operate through altered lipid profiles [68]. This context lends additional biological plausibility to the membrane lipid restructuring observed in phi18:4 virocells in our temporal analysis, suggesting that the host lipid pool may potentially be an active target of phi18:4 infection dynamics. Taken together, the phi18:4 candidate profile suggests a potentially more extensive and acute metabolic remodeling compared to phi18:1, consistent with the distinct replication strategy of its small ssDNA genome.

The inefficient phi38:1 infection yielded two candidates, both significantly enriched relative to uninfected controls. NP-020705 (Level 2) represents a putative natural product analog whose accumulation during inefficient infection warrants further investigation. dl-Alanyl-dl-leucine (Level 3, CMMRT annotation), a dipeptide previously reported to influence bacterial enzymatic regulation [69], may reflect partial protein degradation occurring as the host mounts stress responses in the absence of effective viral reprogramming, consistent with the broader metabolic collapse trajectory observed for phi38:1. While these candidates require experimental validation, their enrichment during inefficient infection contrasts with the energy mobilization signature seen in phi18:1, suggesting that infection outcome may be reflected not only in metabolic trajectory but also in the specific biochemical markers that accumulate.

Collectively, these candidate markers illustrate both the potential and the current limits of computational annotation in virocell metabolomics. Higher-confidence annotations (Levels 1–2) provide tractable biological hypotheses grounded in known biochemistry, while Level 3 candidates generate directionally useful but necessarily speculative interpretations. Re-analysis of this dataset with next-generation annotation tools, including emerging deep learning-based approaches for spectral prediction and structure elucidation, may substantially improve resolution of currently ambiguous candidates and unlock additional mechanistic insight from this rich dataset. Importantly, these candidate biomarkers should not be interpreted as evidence for activation or suppression of specific metabolic pathways. Rather, they represent statistically prioritized metabolites whose known biological associations provide hypotheses for future targeted validation. Determining whether these metabolites reflect altered pathway activity, substrate accumulation, downstream utilization, or regulatory processes will require dedicated biochemical and flux-based studies.

### Pathway-level analysis reveals conserved and infection-specific metabolic restructuring

**Figure 3:**
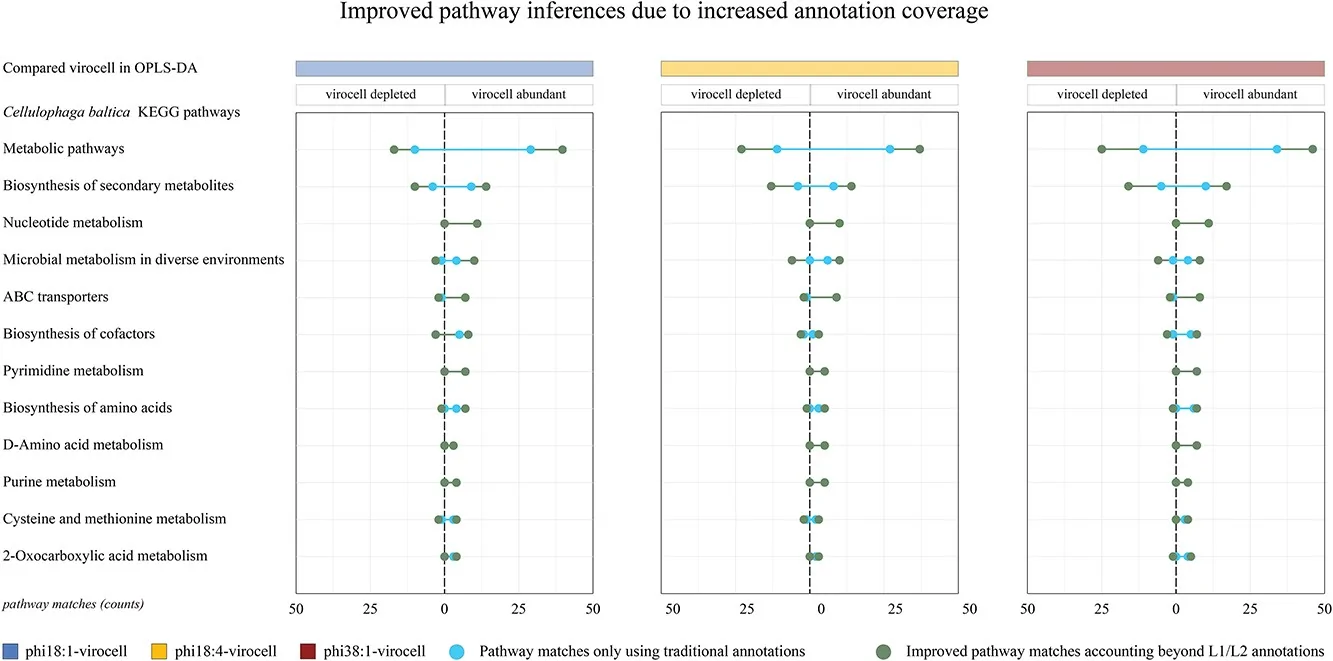
Enhanced annotation coverage improves metabolic pathway inference in virocells. The figure displays the top 12 KEGG pathways for C. baltica that correspond to discriminatory metabolites identified via OPLS-DA, as described in previous sections. Each bar plot shows the number of metabolites mapped to each pathway using two annotation strategies, conventional annotations only and combined annotations that integrate conventional with advanced approaches beyond Levels 1 and 2, as defined in our enhanced framework and indicated in the figure legend. The x-axis denotes the number of metabolites mapped per pathway. Each virocell type (phi18:1, phi18:4, phi38:1) is represented by separate plots, as indicated in the figure legend. Complete pathway match data for all discriminatory metabolites is provided in supplementary workbook S3.

To determine whether deeper metabolite coverage translates into improved functional insight, we next mapped discriminatory metabolites to pathways in the KEGG database [46] (Fig. 3). Because many assignments rely on computational annotations, these pathway associations should be interpreted as hypotheses requiring experimental validation.

Compared to conventional library-only approaches, the expanded framework substantially increased the number of metabolites linked to curated metabolic pathways. Phi18:1 and phi18:4 infections each yielded 266 pathway-associated metabolites, compared with 175 and 178 from traditional annotations. Phi38:1 showed a similar increase, from 197 to 294 pathway-linked metabolites. These gains indicate that broader annotation depth may reveal a more complete picture of infection-driven metabolic restructuring, though database limitations remain substantial [37]. Three major trends emerged from pathway-level interpretation (Workbook S3).

First, deeper annotation expanded coverage of pathways already detectable with conventional analyses. For example, metabolites linked to secondary metabolite biosynthesis increased by 56–75% among enriched features and 43%–65% among depleted features across infections (Fig. 3). Because these pathways are often associated with stress responses, defense mechanisms, and microbial competition [70, 71], improved detection may reveal additional layers of host response to phage pressure. Second, the expanded dataset suggested pathway involvement not previously detected in this system. Ether lipid metabolism, observed only after expanded annotation in phi18:4 and phi38:1 virocells, may indicate membrane remodeling or oxidative stress adaptation during infection [72]. Similarly, glycine, serine, and threonine metabolism appeared in phi18:1 and phi18:4 virocells, implicating pathways linked to redox balance and osmoprotection [68, 72, 73]. While these pathways are well known in microbial physiology, their potential involvement in *C. baltica* infection states represents new system-specific hypotheses that would likely have remained hidden with shallower metabolite coverage. Third, expanded annotation also exposed the limits of current pathway databases. Hundreds of metabolites remained unmapped despite putative identification: 477 in phi18:1, 553 in phi18:4, and 631 in phi38:1 infections, increases of roughly 40%–60% over conventional methods. These unresolved features may include signaling molecules, transient intermediates, or novel analogs, highlighting how infection metabolism may extend beyond currently curated biochemical maps.

Taken together, pathway-level analysis suggests that virocell metabolism involves both conserved shifts, such as increased nucleotide metabolism consistent with viral replication demands, and infection-specific responses. Notably, metabolites mapping to signaling-related KEGG pathways, including quorum sensing-associated and two-component regulatory system pathways were detected exclusively in efficient infections (phi18:1 and phi18:4) and were entirely absent in the inefficient phi38:1 infection (Workbook S3). The detection of metabolites associated with these pathway annotations suggests the possible involvement of structurally related signaling molecules or regulatory intermediates specifically in efficient infection contexts. On this regard, the selective engagement of host regulatory and signaling pathways in efficient infections suggests that productive viral takeover may operate through exploitation or suppression of existing host regulatory networks, a fundamentally different mechanism than direct metabolic hijacking. The absence of these pathway signatures in phi38:1 may therefore reflect not just metabolic inefficiency but a failure to engage host regulatory machinery in a coordinated manner, which may be a defining feature distinguishing efficient from inefficient infection strategies in this system. In contrast, nucleotide metabolism pathways were consistently enriched across all three infections regardless of phage genome type or infection efficiency, representing the most robust conserved pathway-level signature in this dataset. This is biologically expected, as all three phages require nucleotide precursors for genome replication regardless of their distinct replication strategies and provides pathway-level support for the core virocell metabolic program suggested by the discriminatory metabolite analysis above. The consistency of this signal across phages with no shared genes further strengthens its interpretation as a genuine host response to viral replication demand rather than a phage-specific metabolic strategy.

### Temporal metabolic trajectories reveal infection-specific reprogramming strategies

Because phage infection unfolds dynamically, we next examined how host metabolism changes through time during infection of *C. baltica* virocells. Infection kinetics differ substantially among the three phages: phi18:1 exhibits a latent period of ~65 minutes and burst size of 90 [15], phi18:4 shows a shorter latent period of ~45 minutes with burst size 41, and phi38:1 displays a markedly prolonged latent period of approximately 5 h (based on phageFISH measurements) with an estimated burst size of ~39 phages per infected cell [13]. These differences provide a biological framework for interpreting how infection efficiency may shape metabolic trajectories. Because phages differ substantially in latent period, temporal analyses were designed to capture trajectory shape within each phage’s own infection window rather than to compare metabolic states at equivalent absolute timepoints across phages. Therefore, to capture time-resolved metabolic responses, we analyzed longitudinal metabolomic profiles using complementary temporal analyses that identify dominant trends, validate statistical changes, and group metabolites with shared response trajectories [55–57, 74]. Rather than focusing on static differences, this approach allowed us to reconstruct the *shape* of metabolic reprogramming during infection.

**Figure 4:**
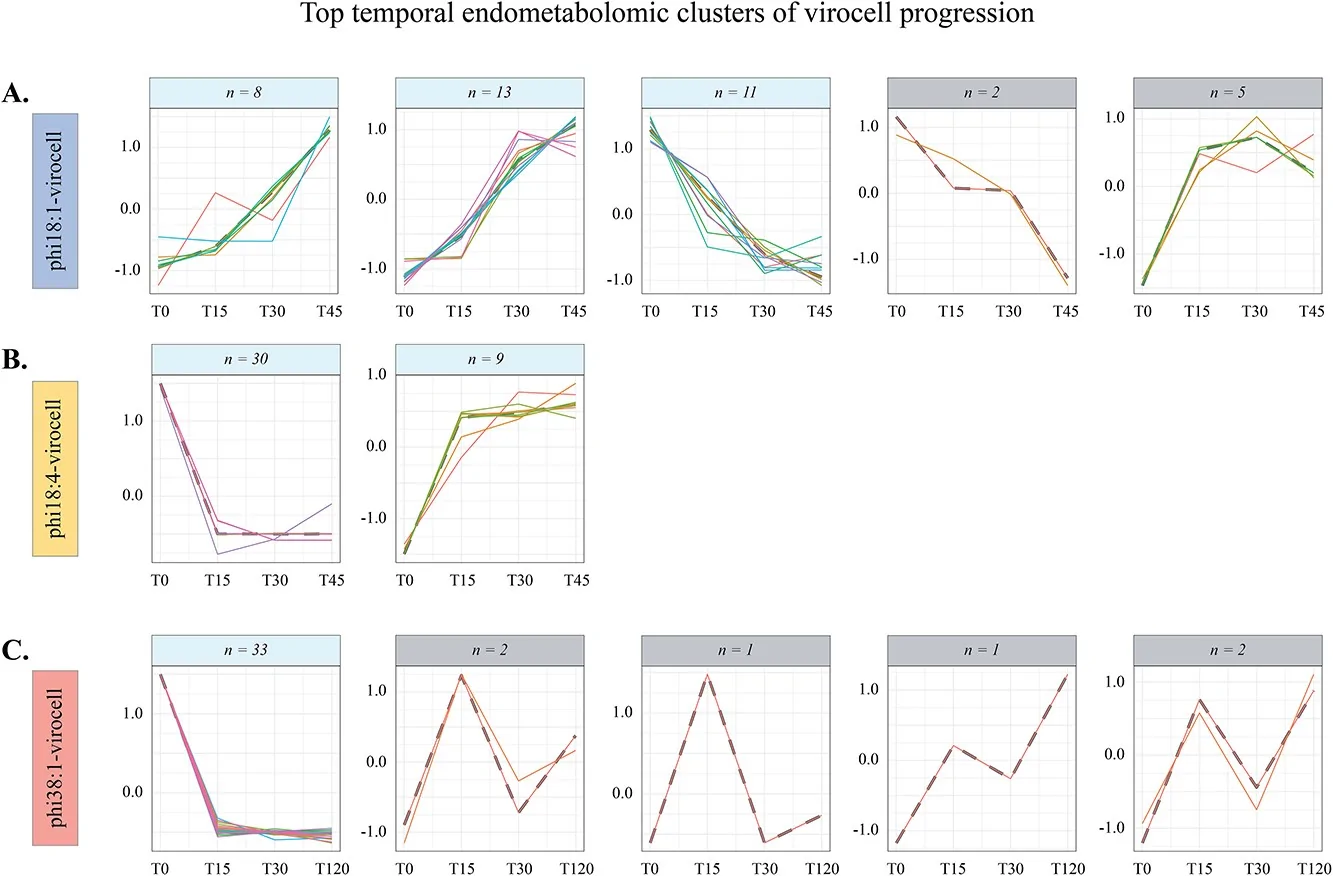
Top temporal clusters of endometabolomic shifts across virocell types. Panels A–C show DTW clustering results of the top 2% significantly changing RP-mode metabolites (n = 39 per virocell type) identified from each PRC analysis: (A) phi18:1, (B) phi18:4, and (C) phi38:1. Metabolites were first selected using PRC analysis, with statistical validation through linear model (LM). Only metabolites passing both criteria were retained for clustering. Each subplot represents a temporal cluster, where each colored line denotes an individual metabolite’s relative change of abundance across time points. Dashed lines represent the average cluster trajectory. Clusters are annotated with their number of members (n), and only those with ≥7 members (≥20% of total metabolites) are highlighted with colored titles and discussed in the main text. Clusters with fewer members are shown with gray titles and included for completeness but not interpreted to avoid overrepresentation of small, potentially spurious trends. Distinct temporal response patterns were observed across virocell types. phi18:1-virocells displayed gradual increasing or decreasing trajectories, phi18:4-virocells showed biphasic behavior, and phi38:1-virocells predominantly exhibited rapid declines. These coordinated metabolite shifts likely reflect virocell-specific infection strategies and metabolic reprogramming dynamics.

We first identified the most temporally responsive metabolites across infections (top 2%, n = 39; Fig. 4), representing dominant biochemical shifts during infection progression. Statistical modeling confirmed that these metabolites changed significantly relative to uninfected controls, supporting their biological relevance (Workbook S2). Clustering of their temporal trajectories revealed distinct infection-specific metabolic dynamics (Workbook S2).

Efficient phi18:1 infection exhibited gradual and coordinated remodeling, with clusters of metabolites showing steady increases (n = 21) or decreases (n = 11) over time. Such progressive shifts suggest controlled metabolic redirection rather than abrupt disruption, consistent with productive infection cycles. Phi18:4 infection, although efficient, showed a different pattern characterized by rapid early changes followed by stabilization. Two dominant trajectories emerged: rapid increases (n = 9) and rapid decreases (n = 30) that plateaued shortly after infection onset. This pattern may reflect fast host takeover followed by metabolic stabilization once viral replication is established. In contrast, the inefficient phi38:1 infection produced a markedly different temporal signature dominated by a sustained decline in metabolite abundance (n = 33) without coordinated increases. This profile suggests a less orchestrated host response, potentially reflecting metabolic collapse or inefficient viral control rather than structured reprogramming.

Expanded metabolite annotation substantially improved biological interpretation of these trajectories. In phi18:1 clusters, conventional annotations explained only one-third of temporal features, whereas expanded annotations suggested additional metabolites including putative N-acetyl amino sugars and peptidoglycan precursors [75]. Their gradual accumulation during infection is consistent with phage-driven cell wall degradation and lytic enzyme activity [76, 77], suggesting that the temporal metabolomics trajectory may be tracking the biochemical progression toward lysis at the molecular level rather than reflecting generalized metabolic perturbation, one of the clearest connections between a specific temporal metabolite pattern and a known mechanistic step in the phage infection cycle observed in this dataset.

In phi18:4 virocells, expanded annotation suggested declines in putative sphinganine and glycerophosphocholine-like metabolites, molecules associated with membrane composition and stress adaptation. This rapid lipid restructuring is consistent with phi18:4’s lipid requirements, where host lipid composition is critical for successful viral replication [15, 68]. The early and rapid nature of these declines, captured in the dominant n = 30 cluster, aligns with phi18:4’s shorter latent period and suggests that lipid remodeling may be among the first coordinated host responses to ssDNA phage takeover. For phi38:1, temporal declines were dominated by putative organic acids and related metabolites, suggesting a broad metabolic shutdown rather than targeted remodeling (Workbook S4).

Interestingly, phi18:4 and phi38:1 displayed broadly similar declining metabolite trajectories despite their fundamental differences in infection efficiency, a pattern consistent with the metabolic drift of phi18:4 virocells toward the phi38:1 metabolic state observed in the global ordination analysis above. This convergence suggests that metabolic suppression may represent a shared host response to phage infection across these two systems, triggered regardless of whether the infection ultimately proceeds productively. What distinguishes the most coordinated infection (phi18:1), and to a lesser extent phi18:4, is the simultaneous occurrence of compensatory metabolic increases alongside suppression of a structured anabolic program that appears absent or insufficient in phi38:1. Efficient infections thus appear characterized by a dual metabolic signature: suppression of host-directed processes alongside coordinated activation of infection-supporting pathways. Inefficient infection, by contrast, produces suppression without compensatory activation, resulting in the net metabolic collapse observed in phi38:1. This distinction between structured reprogramming and unstructured collapse may represent a fundamental metabolic correlate of infection success, and provides a framework for interpreting virocell metabolic states in other phage-host systems.

## Discussion

Our analyses suggest that virocell metabolomes reflect both conserved infection responses and phage-specific metabolic strategies. Across infections, we observed a shared core of discriminatory metabolites, indicating that certain metabolic shifts may represent a generalized host response to viral takeover. At the same time, the large fraction of infection-specific metabolites points to divergent host manipulation strategies across phage types, highlighting the metabolic plasticity of virocells. Furthermore, in this system, infection efficiency appeared closely linked to metabolic coordination. Efficient phages exhibited gradual or structured metabolic transitions, whereas the inefficient phage induced rapid and largely unidirectional metabolic decline. This pattern suggests that successful infections may require sustained metabolic control, whereas inefficient infections may fail to maintain coordinated host reprogramming. Such differences imply that metabolic trajectory shape, rather than endpoint composition alone, may serve as an informative signature of infection strategy. Temporal analyses further indicated that metabolic timing may represent a fundamental dimension of virocell biology. The observation that efficient infections display coordinated temporal remodeling while inefficient infections show abrupt collapse suggests that viral success may depend on maintaining host metabolic homeostasis long enough to support replication. This aligns with the broader concept that virocells represent dynamically regulated metabolic states rather than static infection outcomes. Our findings indicate that viral infection does not simply perturb host metabolism but reorganizes it into structured, pathway-level responses characteristic of the virocell state. By resolving previously hidden metabolites and pathways, we show that infection efficiency is reflected not only in viral replication outcomes but also in the architecture and timing of metabolic reprogramming. These results suggest that metabolome organization itself may serve as a diagnostic feature of successful viral takeover.

These patterns were made accessible by the expanded annotation framework, which increased the fraction of metabolite space available for biological interpretation. While many annotations remain computational and require validation, the ability to observe consistent ecological and infection-dependent patterns across datasets suggests that deeper annotation may substantially improve the hypothesis-generating power of untargeted metabolomics in microbial systems. Importantly, this study does not claim definitive metabolite identification or validated biomarkers. Instead, it demonstrates how integrating complementary analytical tools can expose previously hidden metabolic structure in virocell systems and generate biologically grounded hypotheses for targeted validation.

Throughout this study, we distinguish between robust data-driven observations, the statistical patterns of metabolite trajectories, ordination structure, and discriminatory feature sets and speculative biological interpretations derived from putative annotations. The former represents reproducible findings independent of annotation accuracy; the latter represents hypothesis-generating connections that require experimental validation to confirm. More broadly, this study illustrates how coherent biological signals of viral infection i.e., conserved host responses, phage-specific metabolic strategies, and temporally coordinated reprogramming, can be uncovered by expanding metabolite interpretability beyond conventional untargeted metabolomics workflows. The same strategy can be applied across virus–host systems, environmental microbiology, and host–microbiome interactions, where incomplete metabolite identification often limits interpretation. By enabling pathway-level and temporal insight from complex datasets, such integrative approaches may help reposition untargeted metabolomics as a discovery platform for mechanistic microbial ecology.

## Conclusions

Together, our results suggest that virocell metabolomes encode both conserved host responses and phage-specific metabolic strategies, with infection efficiency reflected not only in metabolite composition but in the temporal coordination of metabolic change. These patterns, made accessible by expanding the interpretable metabolite space, provide a foundation for future experimental validation and ecological exploration of virus-driven metabolic reprogramming. These findings highlight the potential of untargeted metabolomics to move beyond descriptive profiling toward mechanistic insight into virus–host interactions in microbial ecosystems.

## Supplementary Material

Supplementary_material_ycag223

## Data Availability

All data are deposited into public databases and could be accessed via following links: RP LC MS/MS raw data: https://doi.org/10.5281/zenodo.15588023 and, HILIC LC MS/MS raw data: https://doi.org/10.5281/zenodo.15588141. All scripts used for downstream analyses could be accessed via https://github.com/sumuduplus/virocell_metabolomics.
