# Supplementary material for: Metabolome restructuring reveals distinct virocell infection strategies in bacteriophage–host interaction
Source: ISME Commun. 2026 Aug 4;6(1):ycag223. doi: 10.1093/ismeco/ycag223 (PMC13540731; doi:10.1093/ismeco/ycag223)
Supplement: Supplementary file 1 [file supplementary_material_ycag223.zip › Supplementary_document_ycag223.docx]

**Metabolome restructuring reveals distinct virocell infection strategies in bacteriophage–host interaction**

Sumudu Rajakaruna^1,2^, Cristina Howard-Varona^3,4^, Marion Urvoy^3,4^, Roya AminiTabrizi^1^, Christian Ayala-Ortiz^1^, Marissa Gittrich^3,4^, Natalie Solonenko^3,4^, Marie Burris^3^, Courtney Sanderson^3,4^, Cara Noel^3^, Jonathan Leopold^3^, Lillyanna Quillin^3^, Kush Doshi^5^, Lawrence R. Walker^6^, Matthew B. Sullivan^3,4,7^, Malak M. Tfaily^1,2^*

^1^ Department of Environmental Science, University of Arizona, Tucson, AZ, USA

^2^ BIO5 Institute, University of Arizona, Tucson, AZ, USA

^3^Department of Microbiology, The Ohio State University, Columbus, OH, USA

^4^Center of Microbiome Science, The Ohio State University, Columbus, OH, USA

^5^Department of Biochemistry, The Ohio State University, 484 W 12th Ave, Columbus, OH, USA

^6^Analytical and Biological Mass Spectrometry Facility, University of Arizona, Tucson, AZ, USA

^7^Department of Civil, Environmental and Geodetic Engineering, The Ohio State University, Columbus, OH, USA

*Corresponding author: Malak M. Tfaily; [Tfaily@arizona.edu](mailto:Tfaily@arizona.edu)

Supporting Figure 1: Enhanced workflow for untargeted LC-MS/MS-based metabolomics.

Supporting Figure 1: Enhanced workflow for untargeted LC-MS/MS-based metabolomics. The figure illustrates how our enhanced untargeted metabolomics workflow improves upon the traditional/conventional LC-MS/MS approach by expanding metabolite annotation coverage and enabling deeper biological interpretation. Panel A compares traditional and enhanced workflows across four key steps. In panel A(i), both workflows begin with standard sample preparation and LC-MS/MS detection. Panel A(ii) shows the difference in annotation coverage: traditional workflows rely primarily on Levels 1 (pink) and 2 (orange) library-based annotations, resulting in a large proportion of unannotated features (grey). In contrast, our workflow incorporates multi deep-learning based annotation tools that produce Level 2+ annotations (blue), significantly increasing the number of confidently annotated metabolites. Panel A(iii) highlights how this expanded coverage enables more advanced data analyses, including ordination, discriminatory analyses, temporal biosignature detection, biomarker discovery, and pathway inferences. The color gradient represents increasing analytical and biological inference complexity. Panel A(iv) demonstrates a schematic representation of how improved annotation and analysis could lead to more comprehensive biological interpretation, reflected by a larger inner circle of well-characterized metabolites. Panel B provides a decision framework for selecting annotation strategies and analytical tools based on the chromatographic method and study goals. This breakdown highlights the tools used in our workflow CMMRT (1), SIRIUS (2), as well as potential other annotation approaches such as GNPS (3) and MACARRoN (4) towards furthering level 2+ annotations.

Supporting Figure 2: Overview of the analytical workflow employed.

Supporting Figure 2: Different data analytical approaches employed in this study. Raw data obtained for RP and HILIC datasets were independently preprocessed and analyzed using ordination, discrimination, and temporal analysis. The results of discriminatory analyses were combined towards obtaining detailed cross comparison, biomarker and pathway analyses. Specifics of each approach including the tools employed are depicted within each box.

Supporting Figure 3: Multi deep-learning based annotation integration workflow for enhanced LC-MS/MS metabolomics coverage.

Supporting Figure 3: Multi deep-learning based annotation integration workflow for enhanced LC-MS/MS metabolomics coverage. Following in-source fragment filtering, data from each mode proceeded through a tiered annotation process spanning Level 1, Level 2, Level 2.1, and Level 3 annotations, with cumulative coverage depicted at each stage. Level 2.1 annotations were binned as high confidence level 3 annotations for all downstream explanations. Custom curation criteria used for CMMRT multi-hit annotations in the RP dataset are depicted in the flowchart callout (C). The flowchart outlines the prioritization logic applied to probabilistic candidates based on exclusion of synthetic compounds, retention time matching (as 1.1/1.2), presence in KEGG, biological relevance, and chemical class consistency. Annotations that remained unresolved after filtering were excluded from Level 2.1 assignments. Annotation coverage at each step is shown for RP (C) and HILIC (D) modes, with pie charts representing the percentage breakdown of annotations at each level. Unannotated features are shown in grey, while Level 1 (pink), Level 2 (green), Level 2.1 (orange), and Level 3 (blue) annotations highlight the cumulative improvement of the integrated pipeline. Notably, the RP dataset reached 89.95% total annotation coverage, while HILIC achieved 25.55%.

Supplementary Table 1: different detection and analytical platforms used in virocell metabolomics

| Host | Experimental setup | # of metabolites/peaks used | Metabolite Detection Methods used | Main statistical approach used | Ref |
| --- | --- | --- | --- | --- | --- |
| *Sulfitobacter sp. 2047* | Two phages in artificial seawater | 82 metabolites | LC-MS/MS in both positive and negative modes | P ⩽0.05 and ⩾1.5-fold change | 5 |
| *Pseudomonas aeruginosa* | minimal medium with 6 lytic phages | From 918 P. aeruginosa assigned metabolites, 518 metabolites were used | negative mode flow injection-time-of-flight mass spectrometry | P-value ⩽0.05 and abs (log2(fold change) >0.5 | 6 |
| *Synechococcus sp.* | infected with the cyanophage S-SSM5 and co-cultured with the protist Oxyrrhis marina | 264 metabolites | HILIC LC-MS/MS | Log10FC ≥ 0.1 and FDR corrected t,test p. value ≤ 0.05 | 7 |
| *Pseudomonas aeruginosa* | bacterium was engineered to overexpress the phage protein gp70.1 | 49 metabolites | Nuclear magnetic resonance (NMR) spectroscopy | PCA, PLS-DA with VIP scores | 8,9 |
| *Dinoroseobacter shibae DFL12* | Bacterium was infected with 4 diverse Roseobacter phages | 2302 peaks* | LC-MS/MS in both positive and negative modes | Student’s T-tests and OPLS-DA with cutoffs P ⩽0.05 and VIP ⩾1.0 | 10 |

* Metabolites with MS score > 0.6 were used for pathway analyses (n=232)

Supplementary Table 2: OPLS-DA model performances

| **Model** | **R²X(cum)** | **R²Y(cum)** | **Q²(cum)** | **RMSEE** | **pR²Y** | **pQ²** |
| --- | --- | --- | --- | --- | --- | --- |
| phi18:1-RP | 0.462 | 0.998 | 0.994 | 0.0212 | 0.001 | 0.001 |
| phi18:4-RP | 0.577 | 0.996 | 0.989 | 0.0333 | 0.001 | 0.001 |
| phi38:1-RP | 0.598 | 0.999 | 0.997 | 0.0142 | 0.001 | 0.001 |
| phi18:1-HILIC | 0.332 | 0.902 | 0.722 | 0.165 | 0.014 | 0.001 |
| phi18:4-HILIC | 0.331 | 0.939 | 0.808 | 0.132 | 0.021 | 0.001 |
| phi38:1-HILIC | 0.245 | 0.953 | 0.827 | 0.114 | 0.001 | 0.001 |

References cited

1. García CA, Gil-de-la-Fuente A, Barbas C, Otero A. Probabilistic metabolite annotation using retention time prediction and meta-learned projections. J Cheminformatics. 2022 Jun 7;14(1):33. doi:10.1186/s13321-022-00613-8 PubMed PMID: 35672784; PubMed Central PMCID: PMC9172150
2. Ludwig M, Fleischauer M, Dührkop K, Hoffmann MA, Böcker S. De Novo Molecular Formula Annotation and Structure Elucidation Using SIRIUS 4. Methods Mol Biol Clifton NJ. 2020;2104:185–207. doi:10.1007/978-1-0716-0239-3_11 PubMed PMID: 31953819.
3. Aron AT, Gentry EC, McPhail KL, Nothias LF, Nothias-Esposito M, Bouslimani A, et al. Reproducible molecular networking of untargeted mass spectrometry data using GNPS. Nat Protoc. 2020 Jun;15(6):1954–91. doi:10.1038/s41596-020-0317-5
4. Bhosle A, Bae S, Zhang Y, Chun E, Avila-Pacheco J, Geistlinger L, et al. Integrated annotation prioritizes metabolites with bioactivity in inflammatory bowel disease. Mol Syst Biol. 2024 Mar 11;20(4):338–61. doi:10.1038/s44320-024-00027-8 PubMed PMID: 38467837; PubMed Central PMCID: PMC10987656.
5. Ankrah NYD, May AL, Middleton JL, Jones DR, Hadden MK, Gooding JR, et al. Phage infection of an environmentally relevant marine bacterium alters host metabolism and lysate composition. ISME J. 2014 May;8(5):1089–100
6. De Smet J, Zimmermann M, Kogadeeva M, Ceyssens PJ, Vermaelen W, Blasdel B, et al. High coverage metabolomics analysis reveals phage-specific alterations to Pseudomonas aeruginosa physiology during infection. ISME J. 2016 Aug;10(8):1823–35.
7. Howard-Varona C, Roux S, Bowen BP, Silva LP, Lau R, Schwenck SM, et al. Protist impacts on marine cyanovirocell metabolism. ISME Commun. 2022 Dec 1;2(1):94.
8. Zhao X, Shen M, Jiang X, Shen W, Zhong Q, Yang Y, et al. Transcriptomic and Metabolomics Profiling of Phage–Host Interactions between Phage PaP1 and Pseudomonas aeruginosa. Front Microbiol [Internet]. 2017 Mar 30 [cited 2025 May 2];8. Available from: https://www.frontiersin.orghttps://www.frontiersin.org/journals/microbiology/articles/10.3389/fmicb.2017.00548/full
9. Zhao X, Chen C, Jiang X, Shen W, Huang G, Le S, et al. Transcriptomic and Metabolomic Analysis Revealed Multifaceted Effects of Phage Protein Gp70.1 on Pseudomonas aeruginosa. Front Microbiol. 2016 Sep 26;7:1519.
10. Min Jin, Lanlan Cai, Longfei Lu, Meishun Yu, Rui Zhang, Combined metabolomic and genomic analyses reveal phage-specific and infection stage-specific alterations to marine Roseobacter metabolism, ISME Communications, Volume 5, Issue 1, January 2025
